# Ecotoxicity of Diazinon and Atrazine Mixtures after Ozonation Catalyzed by Na^+^ and Fe^2+^ Exchanged Montmorillonites on *Lemna minor*

**DOI:** 10.3390/molecules28166108

**Published:** 2023-08-17

**Authors:** Amina Benghaffour, Abdelkrim Azzouz, David Dewez

**Affiliations:** 1 NanoQAM, Department of Chemistry, University of Quebec at Montreal, Montreal, QC H3C 3P8, Canada; 2 École de Technologie Supérieure, Montreal, QC H3C 1K3, Canada

**Keywords:** diazinon, atrazine, adsorption, ozonation, *Lemna minor*, toxicity

## Abstract

The toxicity of two pesticides, diazinon (DAZ) and atrazine (ATR), before and after montmorillonite-catalyzed ozonation was comparatively investigated on the duckweed *Lemna minor*. The results allowed demonstrating the role of clay-containing media in the evolution in time of pesticide negative impact on *L. minor* plants. Pesticides conversion exceeded 94% after 30 min of ozonation in the presence of both Na^+^ and Fe^2+^ exchanged montmorillonites. Toxicity testing using *L. minor* permitted us to evaluate the change in pesticide ecotoxicity. The plant growth inhibition involved excessive oxidative stress depending on the pesticide concentration, molecular structure, and degradation degree. Pesticide adsorption and/or conversion by ozonation on clay surfaces significantly reduced the toxicity towards *L. minor* plants, more particularly in the presence of Fe(II)-exchanged montmorillonite. The results showed a strong correlation between the pesticide toxicity towards *L. minor* and the level of reactive oxygen species, which was found to depend on the catalytic activity of the clay minerals, pesticide exposure time to ozone, and formation of harmful derivatives. These findings open promising prospects for developing a method to monitor pesticide ecotoxicity according to clay-containing host-media and exposure time to ambient factors.

## 1. Introduction

Pesticides are organic compounds used for pest control purposes and classified into several groups depending on the pest organisms targeted. Pesticides are hardly (bio)degradable, and their presence has been significantly increased in freshwater environment due to agricultural runoffs contaminated by pesticide wastes. The use of pesticides has detrimental effects on freshwater reservoirs serving as drinking water, and it has become a major environmental and public health issue. In natural ecosystems, non-target aquatic organisms can accumulate pesticide doses causing toxicity impacts on major cell physiological processes (e.g., photosynthesis, respiration, enzymatic activities, macromolecules biosynthesis), leading to growth inhibition and eventuality death [1,2,3].

Among extensively used pesticides contaminating surface freshwaters, diazinon (DAZ) and atrazine (ATR) are well known as being hazardous compounds acting via different toxicity mechanisms. DAZ is a neurotoxic organophosphorus insecticide inhibiting the acetylcholinesterase activity, and ATR is an herbicide hindering the photosynthetic electron transport and the growth of plants [4,5,6]. Any attempt to preserve the freshwater quality unavoidably would induce the degradation of these pesticides into derivatives exhibiting chemical and toxicological properties [7,8]. The latter needs to be investigated for water remediation technologies and toxicity risk assessment.

Pesticides removal from water can be performed by chemical treatments such as photolysis, hydrolysis, and oxidation [9,10]. Ozone-based advanced oxidation processes (AOPs) are particularly efficient methods for achieving high removal rates of organic pollutants [11]. Ozonation is a complex process that should involve a variety of transformations such as tautomerization, isomerization, protonation and dimerization, and others [12]. Pesticide molecules are oxidized by generated ozone molecules and/or hydroxyl radicals depending on the pH into various oxidized derivatives. Nonetheless, total mineralization into harmless carbon dioxide (CO_2_), water, and other inorganic oxides, if any, is seldom attained by ozonation unless using efficient catalysts.

Owing to their adsorption capacity that reduces ozone consumption, solids catalysts already turned out to be much more effective and convenient as compared to their soluble counterparts that often induce contamination of the treated waters. The use of widely available, low cost, and recyclable clay minerals is particularly interesting when targeting ‘’green’’ ozone-based water treatments. Clay catalysts display expandable structure without pore size limitation and coagulation-flocculation capacity that allow easy recovery after settling. Among clay catalysts, montmorillonite ion-exchanged by alkaline metal and transition metal was found to exhibit high catalytic activity in the ozonation of organic molecules [13,14,15,16,17,18,19,20,21,22].

Like in nature, total mineralization of the parent organic molecules and intermediates cannot be achieved by incomplete oxidation, low doses of oxidizing species, and/or short exposure times. The persistence of these derivatives in nature is usually proportional to their oxidation level. This explains the fact that highly oxidized intermediates and short chain acids are quite refractory to ozone even after total degradation of the parent molecule. Their mere presence in waters is a new source of ecotoxicological effects that is worthy of being investigated. Since DAZ and ATR oxidation can also lead to new hazardous derivatives with toxicological properties, it is crucial to monitor the toxicity of their ozonation mixtures with an appropriate testing method. At this juncture, using a sensitive organism model for such a toxicological evaluation will be useful in investigating the effect of the oxidation grade of different pesticide-containing solutions obtained after different ozonation times. Among such sensitive organism models, an aquatic vascular plant, *Lemna minor* (Lemnaceae), and microcrustacean *Daphnia magna* have widely been used in several pollutant toxicity testing studies, being regarded as reliable ecological bioindicators at the basis of the aquatic food chain [23,24,25,26]. However, *Lemna minor* is known to be much easier to cultivate in laboratory conditions for toxicity testing, and the plants can capture and accumulate rapidly dispersed or dissolved contaminants due to its small structure and high contact surface [27].

In this study, *Lemna minor* population was used for a comparative study of the toxicity before and after catalytic ozonation of two pesticides with different molecular structures and chemical properties, namely diazinon (DAZ) and atrazine (ATR). This was achieved in so-called SIS medium, i.e., aqueous solution prepared according to the Swedish Institute for Standards. Like any organic molecule bearing chemical functions, DAZ and ATR will interact with the aqueous media and dispersed species according to the pH. All these interactions are expected to influence their adsorption and ozonation, and the latter, in turn, should also modify their ecotoxicity. This was the main objective of this research.

In this regard, the effects of clay catalysts on the ozonation advancement in time on different toxicity biomarkers related to cell growth and physiological state of plant were considered. This study is expected to contribute to a better understanding of how the oxidative degradation of pesticides can modify the toxicological properties of their host-media and, therefore, the environmental impact. The monitoring relevance of using *Lemna* toxicity testing was also discussed herein.

## 2. Results and Discussion

### 2.1. Intrinsic Effect of Pesticide on Plant Growth

**Figure 1:**
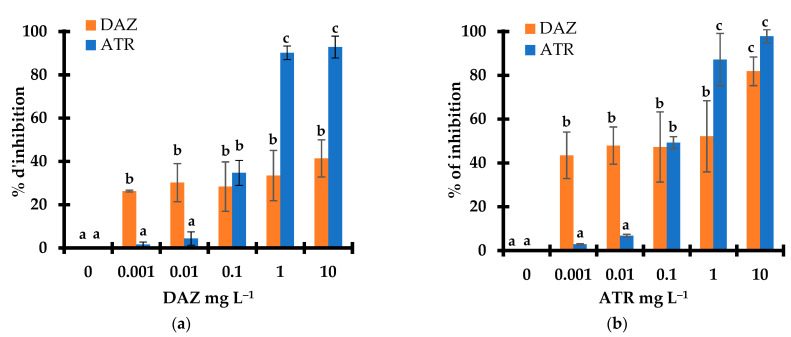
Effect of pesticide concentration on growth inhibition of *Lemna minor* based on fronds number (**a**) and fresh weight (**b**) after 7 days of exposure. Triplicates measurements were achieved for all pesticides concentrations including the pesticide-free control sample (0 mg L^−1^). The control has an original value of fresh weight of 99.5 mg and an average number of 63.5 fronds. Different letters used as symbols account for different data significances at *p* < 0.05. Data with similar significance are marked with similar symbols (Characters).

Pesticides are synthetic organic molecules that act as alien chemical species with intrinsic toxicity for living species growths and metabolisms, even before degradation. The toxicity of different concentrations of DAZ and ATR ranging from 0.001 to 10 mg L^−1^ on *L. minor* after 7 days exposure was first evaluated through the inhibition grade of two complementary growth parameters, namely the frond number and fresh weight. A first overview of the results obtained revealed slightly lower values of the inhibition grade of the frond number (Figure 1a) as compared to the fresh weight (Figure 1b) upon exposure to both pesticides. This accounts for a stronger negative effect on the biomass growth as compared to frond multiplication.

Similar increasing tendency of the inhibition grade for both parameters was observed with increasing pesticide concentration. This increase was much more accentuated for ATR as compared to DAZ. This indicates a stronger toxicity of ATR towards *L. minor*, in agreement with its herbicide action and harmfulness against plants. The *L. minor* fronds showed no signs of deterioration, necrosis, or chlorosis after 7 days of exposure.

Based on the fresh weight data, the half-maximal effective concentration (EC50) was estimated to be of 0.85 and 0.045 mg L^−1^ for DAZ and ATR, respectively. This higher toxicity of ATR was already explained in terms of its molecular structure. The latter is specifically tailored for ATR to act as an herbicide unlike diazinon that rather exhibits insecticide features [28,29,30,31]. This EC50 value of ATR is slightly lower than that reported for *L. minor* but approximately twice lower as compared to that registered for other living species such as *L. gibba* (0.089 mg L^−1^) [25,32,33,34,35].

This indicates a higher sensitivity of *L. minor* to atrazine, thereby justifying the choice of such a plant species as a reliable bioindicator for accurate assessment of pesticide ecotoxicity. Here, even in trace amounts, ATR is supposed to affect photosynthesis with detrimental effect on plant growth. It acts as uncouplers to prevent oxidative phosphorylation and formation of the proton gradient. This should involve ATR binding to the electron acceptor protein, thus inhibiting electron transport [34,36]. Therefore, deeper insights in the pesticide effects on photosynthetic pigments and the level of reactive oxygen species (ROS) are expected to provide valuable data for elucidating not only the intrinsic toxicity pathway of ATR but also of its oxidized derivatives. During the 7-day exposure of *L. minor*, pesticides can not only interact with oxidized species but also and instantly with ROS before their fast disappearance.

### 2.2. Change in Photosynthetic Pigments

**Figure 2:**
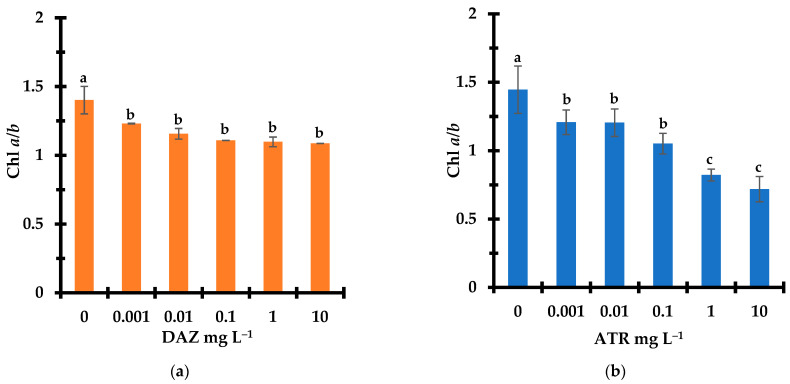
Effect of DAZ (**a**) and ATR (**b**) concentration on (Chl *a*/*b*) ratio of *Lemna minor* after 7 days exposure. Triplicates measurements were achieved for all pesticides concentrations including the pesticide-free control sample (0 mg L^−1^). Different letters used as symbols account for different data significances at *p* < 0.05. Data with similar significance are marked with similar symbols (Characters).

Photosynthesis is an essential process for plants growth and can be altered directly or indirectly by several pollutants. The latter can affect the very action of the photosynthetic pigments and their distribution, often reflected by detectable changes in the contents of chlorophylls *a* and *b* (Chl *a* and Chl *b*). That is why the ratio Chl *a*/*b* was used as a general biomarker of chlorophylls biosynthesis and of chlorophyll–proteins complexes. The Chl *a*/*b* ratio was found to decrease from its starting ratio of ca. 1.35–1.40 for the control sample with increasing concentration of both pesticides from 0 to 10 mg L^−1^, indicating changes in the distribution of the photosynthetic pigments (Figure 2).

More pronounced and continuous Chl *a*/*b* decrease down to 0.40–0.45 was noticed within the entire ATR concentration range (0–10 mg L^−1^) as compared to DAZ. The latter showed a weaker Chl *a*/*b* decrease down to a plateau at approximately 1.2 in the DAZ concentration range of 0.1–10 mg L^−1^. This suggests detrimental effects of both pesticides on the photosynthetic pigments, more pronounced towards Chl *a* as compared of Chl *b*, and a stronger toxicity of ATR as compared to DAZ.

### 2.3. ROS Level under Pesticide Stress

**Figure 3:**
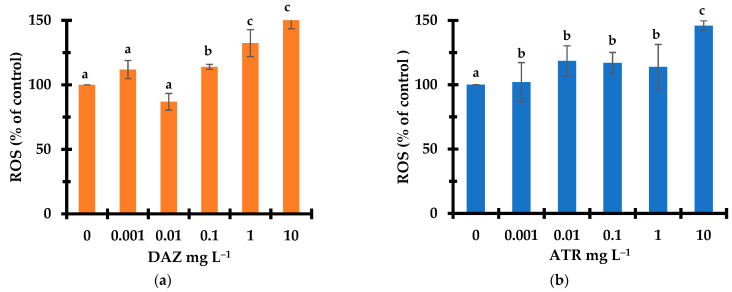
Effect of DAZ (**a**) and ATR (**b**) concentration on *Lemna minor* ROS level after 7 days exposure. Triplicates measurements were achieved for all pesticides concentrations including the pesticide-free control sample (0 mg L^−1^). The control has a relative fluorescence intensity value of 435. Different letters used as symbols account for different data significances at *p* < 0.05. Data with similar significance are marked with similar symbols (Characters).

In nature, *L. minor* is continuously exposed to oxidative stress due to the accumulation of ROS during different metabolic processes [37]. The plant has a natural capacity to trigger protective mechanisms involving antioxidant compounds and enzymatic activities [38]. The presence of oxidizing agent and oxidant precursors such as ozone, UV radiation, unusual acidity or alkalinity, heavy metals and organic pollutants can induce excessive ROS level [39,40]. The latter would readily affect cell components by oxidizing essential macromolecules, such as lipids, proteins, and nucleic acids [41,42]. In this study, exposure to increasing pesticide concentrations in the range of 0–10 mg L^−1^ induced a visible increase in ROS level for both pesticides (Figure 3).

After 7 days of exposure, ROS level in *L. minor* plants increased by 54–55 and 45–46% with increasing concentrations of DAZ and ATR, respectively. The rise of excess ROS induced by the pesticide with respect to the control sample explains the inhibition intensification of the plant growth. This confirms the key-role of ROS in the toxicity of both pesticides, in agreement with the previous expectation of ROS-based alteration of the metabolism pathway. This different ROS level must be due to different mechanisms in the oxidative stress between DAZ and ATR, and contrasts with their reversed toxicity sequence. The fact that DAZ induced higher ROS level at the highest concentrations (1 and 10 mg L^−1^), but lower toxicity as compared to ATR suggests the occurrence of different mechanisms pathways and different type and/or distribution of ROS.

A possible explanation resides in the fact that DAZ toxicity is assumed to induce an oxidative stress in the cytosol. ATR would rather trigger the oxidative stress in the chloroplast through the alteration of chlorophyll–proteins complexes in the photosystems and by blocking the electron flow in the photosystem II [43]. The induction of ROS such as O_2_^•−^ radical, H_2_O_2_ and ^•^OH can result from a series of reactions starting with the interaction between chlorophyll molecules and oxygen, forming singlet oxygen (^1^O_2_) [44]. Other possible explanations could involve the role of the early physical-chemical interactions between the pesticide molecules and each of the key-actors in the *L. minor* autoprotective system, even before the oxidative stress is triggered. This remains to be elucidated by deeper insights.

### 2.4. Effect of Adsorption and Catalytic Ozonation

A comprehensive strategy resides in investigating the role of both adsorption and oxidation as simulated by ozonation in the presence of clay-materials (2 g L^−1^) in the toxicity changes on *L. minor* using the highest concentration of pesticide (10 mg L^−1^). Investigating the effect of ozonized DAZ and ATR reaction mixture is a useful approach for demonstrating that, in nature, pesticide ecotoxicity evolves in time, according to the host media. It also provides evidence that any attempt to oxidative treatments of pesticide-containing waters can contribute to this toxicity evolution. In nature, pesticides are usually spread on soils supposed to contain clays, but they can also be dispersed in larger areas by runoff waters.

**Figure 4:**
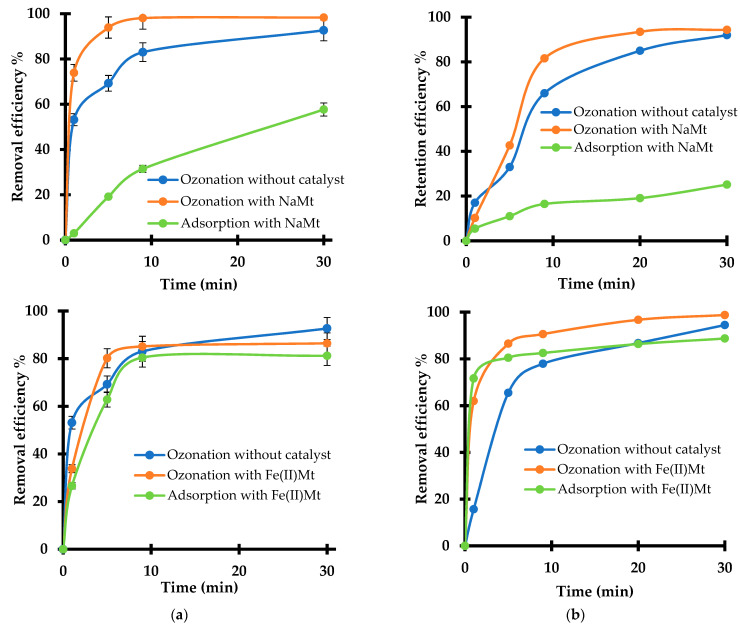
Removal efficiency of DAZ (**a**) and ATR (**b**) by adsorption and ozonation in the presence of 2 g L^−1^ NaMt (upper panel) and Fe(II)Mt (down panel). Sample volume = 25 mL. The relative peak area (A/A0) was calculated as the instant/initial HPLC-UV peak area ratio of DAZ or ATR. This ratio allows expressing the adsorption efficiency as: (1 − A/A0) × 100%. Triplicates measurements were achieved including the non-adsorbed and non-ozonized pesticide solution taken as the starting control sample (10 mg L^−1^).

They can undergo physical-chemical interactions such as adsorption in solid particles and oxidation in both adsorbed and dispersed states in both soil- and water-containing clays. As a general feature, ozonation produced higher removal efficiency than adsorption for both pesticides in the presence of 2 g L^−1^ of NaMt or Fe(II)Mt (Figure 4).

This clay concentration is regarded as being close to the critical coagulation concentration of clay minerals [45], being optimal for high catalytic activity in the ozonation of organic pollutants [14,17,18,20,21,22]. The difference between both removal rates was found to be in the same magnitude order as the pesticide conversion by non-catalytic ozonation. This result provides evidence that adsorption is a key-step in clay-catalyzed ozonation. The general increase of the removal efficiency in time for both pesticides and both catalysts indicates that the adsorption and/or diffusion of the reactant (ozone and pesticide) appears as kinetic-controlling steps.

NaMt gave pesticide conversion rates of up to 97–98% for DAZ after only 10–20 min ozonation, similar to those obtained with Fe(II)Mt but after longer ozonation (30 min). This accounts for slower initial ozonation for Fe(II)Mt that contrasts with its much higher adsorption rates for DAZ (80–81% after 10 min) and ATR (up to 83–86% after 30 min) registered in the presence of Fe(II)Mt as compared to NaMt (58% after 30 min and 23–25%, respectively). A possible explanation should consist in the fact that massive adsorption of pesticides induces the appearance of diffusion hindrance. This must be due to a “sandwiching” effect of bivalent Fe^2+^ cations shared by next neighboring clay sheets that reduces the swelling capacity of Fe(II)Mt [46].

The higher removal by ozonation as compared to adsorption and the much lower contribution of adsorption on NaMt as compared to Fe(II)Mt must be due to specific clay–pesticide and clay–clay interactions. The latter should be strongly influenced by the unavoidable pH decrease during ozonation. Indeed, various clay–clay, pesticide–clay, pesticide–ozone, clay–ozone, and more particularly those involving silanol groups (-Si-O-H) can take place according to the pH level attained [16,47].

### 2.5. Toxicity Changes upon Pesticide Adsorption

**Figure 5:**
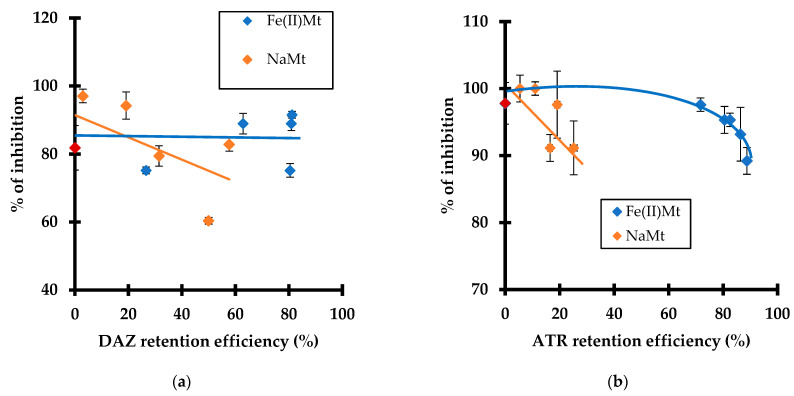
Effect of the retention efficiency (%) of DAZ (**a**) and ATR (**b**) with 2 g L^−1^ of Fe(II)Mt and NaMt on the inhibition grade (%) of *Lemna minor*. Triplicates measurements were achieved including the non-adsorbed and non-ozonized pesticide solution taken as the starting control sample (red symbol, 10 mg L^−1^).

Pesticide impacts on plant growth are expected to evolve specifically in time according to the molecular structure of the organic molecules, external environmental factors, and features of the host-media. Therefore, depending on the starting intrinsic toxicity, the progressive pesticide degradation should significantly modify the global impact of a given pesticide through changes in the intermediate distribution. In this study, the toxicity of DAZ and ATR on *L. minor* plants was evaluated after different times of adsorption and catalytic ozonation in SIS medium followed by pH adjustment to 6.5. The results revealed a decrease in the growth inhibition rate with increasing retention efficiency for both DAZ and ATR on NaMt but almost no modification on Fe(II)Mt (Figure 5).

This decrease was somehow expected since pesticide adsorption on NaMt reduced both the amount of residual non-adsorbed molecules and persistent toxicity towards *L. minor*. In contrast, the presence of Fe(II)Mt paradoxically induced almost constant inhibition grade. This can be explained by the fact that any fluctuations of the inhibition grade are shaded by the higher pesticide removal efficiency through adsorption on Fe(II)Mt as compared to NaMt.

### 2.6. Toxicity Induced by Pesticide Degradation

**Figure 6:**
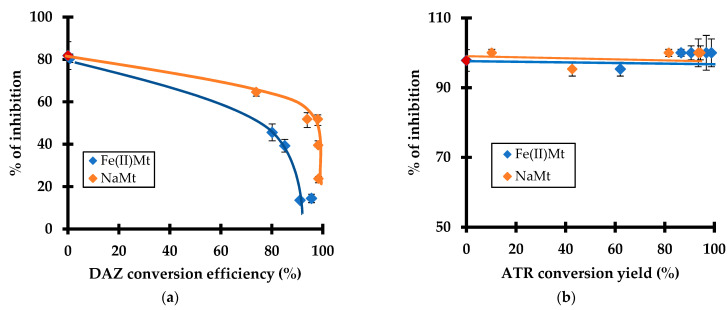
Effect of the conversion efficiency by ozonation (%) of DAZ (**a**) and ATR (**b**) with 2 g L^−1^ of Fe(II)Mt and NaMt on the inhibition grade (%) of *Lemna minor*. Triplicates measurements were achieved including the non-adsorbed and non-ozonized pesticide solution taken as the starting control sample (red symbol, 10 mg L^−1^).

As expected, increasing DAZ conversion by ozonation resulted in decreasing toxicity reflected by a depletion of the inhibition grade. This depletion was more pronounced in the presence of Fe(II)Mt as compared to NaMt (Figure 6a). This is due to a progressive decay of the residual toxicity towards *L. minor* because of depleting amount of residual non-adsorbed molecules of DAZ. This effect seems to be shaded by the higher toxicity of ATR (Figure 6b) and/or the progressive appearance of more harmful ATR derivatives as compared to those of DAZ.

Among ATR derivatives, desethyl-atrazine, desisopropyl-atrazine, desethyl-desisopropyl-atrazine, and hydroxyderivatives of these compounds, namely 2-hydroxyatrazine, 2-hydroxy-desethylatrazine, 2-hydroxy-desisopropylatrazine and 2-hydroxy-desethyl-desisopropylatrazine were already identified [25]. These intermediates are hydroxylated and dealkylated compounds [48]. Some degradation products may be more toxic than the parent compound [49]. The most pronounced toxicity was noticed with desethyl-atrazine, desisopropyl-atrazine, and desethyl-desisopropyl-atrazine [50].

DAZ degradation was rather found to generate diazoxon, hydroxydiazinon, hydroxyethyl derivative of diazinon, diazinon methyl ketone and 2-hydroxydiazinon. Most of these intermediates turned out to be less toxic as compared to the parent diazinon molecule [51]. In contrast, diazoxon displayed the highest toxicity [52,53] even for fish [54].

### 2.7. Effect of Pesticide Removal on ROS Level

**Figure 7:**
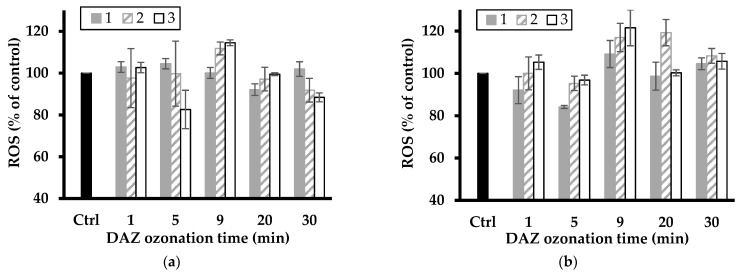
Change in ROS level of *L. minor* in DAZ ozonized mixture in the presence of NaMt (**a**) and Fe(II)Mt (**b**) catalysts (1, 2 and 3 g L^−1^). The data represent triplicate measurements after plant exposure for 7 days including the control sample (Ctrl: *L. minor* in SIS without DAZ ozonized).

**Figure 8:**
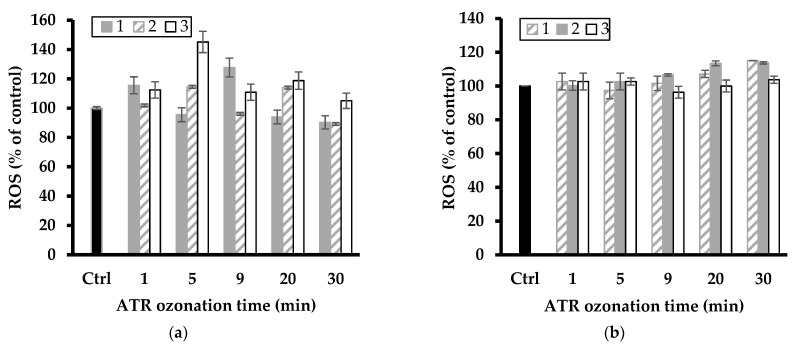
Change in ROS level of *L. minor* exposed to ATR ozonized mixture in the presence of NaMt (**a**) and Fe(II)Mt (**b**) catalysts (1, 2 and 3 g L^−1^). The data represent triplicate measurements after plant exposure for 7 days including the control sample (Ctrl: *L. minor* in SIS without ATR ozonized).

A first overview of the data illustrated by Figure 7 and Figure 8 showed fluctuations in ROS level with increasing ozonation time, suggesting toxicity changes with the pesticide degradation level. The most plausible explanation resides in the successive formation of various intermediates that induce different oxidative stress on *L. minor*. These fluctuations also varied with increasing clay catalyst concentration in the ozonation mixture and were more pronounced for the relatively high catalyst concentrations of 2–3 g L^−1^.

The latter gave the highest increase in ROS level up to ca. 112 and 116% (with NaMt), and 118 and up to 121% (with Fe(II)Mt) after 9 min ozonation of DAZ mixture as compared to the intrinsic ROS value of the control sample (100%). The slight ROS level from ATR agrees with its higher growth inhibition induced by ozonation on *L. minor* (Figure 1) as compared to NaMt. ATR ozonation gave the highest increase in ROS level up to ca. 113 and 145% (with NaMt) after 5 min ozonation of ATR, and 108 and up to 118% (with Fe(II)Mt) after 20–30 min ozonation as compared to the intrinsic ROS value of the control sample (100%).

This result is of great importance because it demonstrated that the toxicity towards *L. minor* and the ROS level evolve simultaneously. Their evolution varies according to the catalytic activity of the clay minerals and during the progressive oxidative degradation of the pesticides and derivatives. This provides a clear confirmation of the narrow relationship between both factors. A plateau corresponding to a constant ROS level was observed with increasing ATR conversion efficiency by ozonation (Figure 6). This must be due to a shading effect of simultaneous ATR depletion and progressive rise of harmful derivatives, thereby confirming the previous statements.

The distribution of ozonation intermediates appears to strongly depend on the type of ion-exchanged clay minerals. Their common behavior governs the heterogeneous processes of adsorption and ozonation and the extent of the surface contact between the clay catalyst and reactants. The specific feature of the exchangeable cations determines the type of clay–clay and clay–reactant interactions. Unlike in dry media, the particle size of both montmorillonites significantly varies according to charge-compensating cations and the chemical composition of the clay suspension concentration and pH [16,47,55]. These factors will govern the intensity and pathway of the pesticide degradation process, providing evidence that pesticide persistence in nature depends on the type and chemical composition of the host-medium, in agreement with the targeted objective. This finding is also useful to design potential technology based on montmorillonite-catalyzed ozonation for downstream reduction of pesticide ecotoxicity in agriculture wastewaters.

## 3. Materials and Methods

### 3.1. Clay Materials Preparation and Characterization

Fully ion-exchanged Na^+^ and Fe^2+^-montmorillonite-rich materials (NaMt and Fe(II)Mt, respectively) were prepared through purification of a crude bentonite supplied by Sigma-Aldrich (Burlington, MA, USA) according to a procedure described previously [16]. Preliminary characterization through X-ray diffraction (Siemens D5000, CuKα, λ = 1.54051 Å) of both montmorillonites revealed no crystallinity loss. The sharper 001 XRD reflexion of these homo-cationic forms of montmorillonite as compared to the starting bentonite indicated an improvement of the parallel layout of the clay lamellae. Both clay materials were used in adsorption and ozonation tests prior to the toxicity evaluation. The particle size for NaMt and Fe(II)Mt was of 689 nm and 1189 nm in distilled water and of 1107 nm and 3020 nm in SIS media at intrinsic pH, respectively.

### 3.2. Pesticide Stock Solutions

Diazinon (DAZ) as an insecticide and atrazine (ATR) as an herbicide were used for their different chemical and toxicological properties. Commercial DAZ 500E solution (CAS no 333-41-5) with a 500 g L^−1^ concentration was provided by Pr. Philippe Juneau’s laboratory (Department of Biological Sciences, UQAM, Montreal, QC, Canada). ATR (PESTANAL analytical standard, purity ≥ 98.0%, CAS no 1912-24-9) was purchased from Sigma-Aldrich Canada Co (Oakville, ON, Canada).

### 3.3. Adsorption and Ozonation Experiments

**Figure 9:**
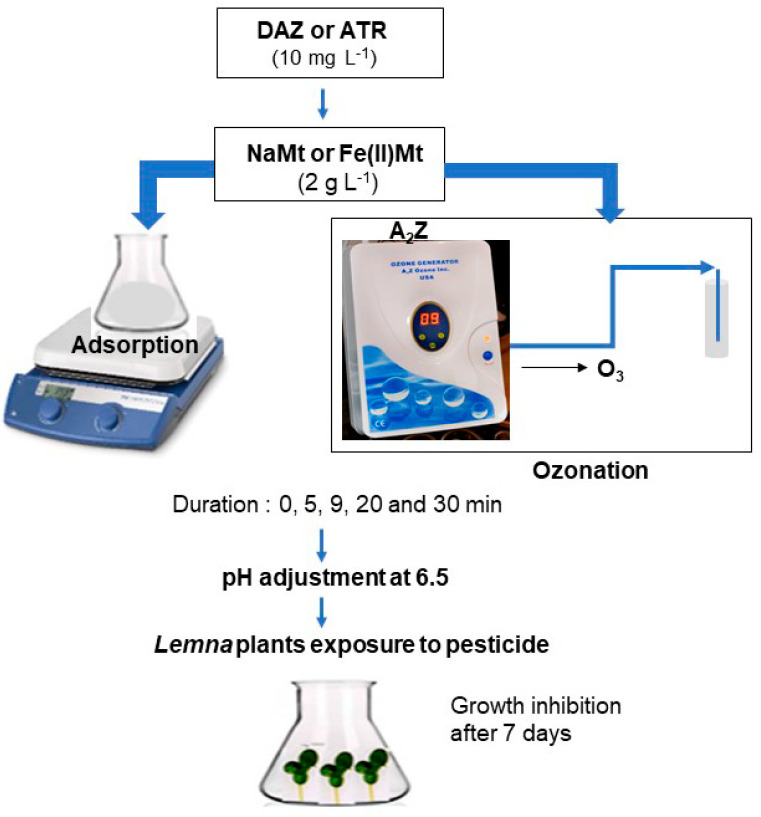
Schematic procedure for investigating the effects of pesticide adsorption and ozonation in the presence of two montmorillonites (NaMt and Fe(II)Mt) on the toxicity towards *Lemna minor*.

Both adsorption and ozonation tests were carried out separately in SIS media without or with added 10 mg L^−1^ pesticide. Adsorption tests were performed by impregnating 2 g L^−1^ of clay adsorbent in 25 mL of 10 mg L^−1^ SIS-pesticide solution under vigorous stirring at room temperature (RT = 25 ± 2 °C) for 1, 5, 9, and 30 min (Figure 9).

In a second step, ozonation tests were performed in glass tubes (28 mm × 115 mm) containing 25 mL of each SIS-pesticide solution and 2 g L^−1^ of clay catalyst under a constant 600 mg h^−1^ ozone bubbling fed by an A2Z generator (A2Z Ozone Inc, Louisville, KY, USA) at room temperature for similar ozonation times. After adsorption and ozonation, the resulting mixtures were centrifugated and the pH was adjusted to 6.5 using 1 N NaOH or HCl. The supernatant of each adsorption or ozonation mixture was analyzed through High-Performance Liquid Chromatography coupled to an ultraviolet-visible detector (HPLC-UV) and then exposed to *Lemna minor* plants for toxicity testing. HPLC-UV analysis was performed using Agilent Technologies model 1290 equipment. HPLC grade methanol (Sigma-Aldrich, Saint Louis, MO, USA) was employed as the mobile phase (Water/Methanol (45/55 *v*/*v*) for DAZ and (80/20 *v*/*v*) for ATR) at an isocratic detection mode. HPLC apparatus used a C18 column (4.6 × 150 mm, 5 µm particle size) at a wavelength detection of 250 nm for DAZ and 220 nm for ATR. Column temperature was fixed at 40 °C and the flow rate was 1 mL min^−1^. The removal efficiency of DAZ and ATR after adsorption or ozonation in the presence of NaMt and Fe(II)Mt were calculated by the equation: (1 − A/A0) × 100%, on the basis of the instant/initial HPLC peak area of the residual pesticide concentration (A/A0) as determined by calibration plots.

### 3.4. Lemna minor Cultivation

Plants of *Lemna minor* were obtained from a culture collection from the Centre St-Laurent’s laboratory of Environment and Climate Change Canada (Montreal, QC, Canada). The cultivation was carried out in accordance with the Organization for Economic Co-operation and Development (OECD) guideline 221 [56]. The plants were grown in an aqueous medium prepared according to the Swedish Institute for Standard (SIS), i.e., a growth medium with the following nutritive chemical composition (in mg L^−1^): NaNO_3_, 85; KH_2_PO_4_, 13.4; MgSO_4_•7H_2_O, 75; CaCl_2_•H_2_O, 36; Na_2_CO_3_, 20; H_3_BO_3_, 1; MnCl_2_•4H_2_O, 0.20; Na_2_MoO_4_•5H_2_O, 0.010; ZnSO_4_•7H_2_O, 0.050; CuSO_4_•5H_2_O, 0.005; Co(NO_3_)2•6H_2_O, 0.010; FeCl_3_•6H_2_O, 0.84, Na_2_-EDTA•2H_2_O, 1.4; MOPS buffer, 490. The medium pH was adjusted to 6.5 ± 0.2, and then filtered through a 0.2-µm filter for experiments under axenic condition. Stock plants were maintained under 24 ± 2 °C, with a photoperiod of 16 h of illumination (light intensity of 100 ± 10 µmol of photons m^−2^ s^−1^) and 8 h of darkness, under a relative humidity of 60 ± 5%.

### 3.5. Lemna Plants Exposure to Pesticide

In a first step, the intrinsic toxicity of DAZ and ATR was assessed using five nominal concentrations of each pesticide previously prepared in SIS medium: 0.001, 0.01, 0.1, 1, 10 mg L^−1^ and then exposed to three triple-fronded *Lemna minor* for 7 days. In a second step, a 20 mL volume of each supernatant of the adsorption and ozonation mixtures was put in contact in a 50 mL Erlenmeyer with three triple-fronded *L. minor* plants without any lesions or chlorosis. Measurements of the growth rate, the pigment content, and the level of ROS were achieved after 7 days exposure to the same light and temperature conditions as the stock culture.

### 3.6. Growth Inhibition

After 7 days, the number of fronds and fresh weight were estimated in each flask and compared to blank samples consisting of non-exposed plants. The growth rate was calculated based on the measured fresh weight or fronds number using the Equation (1), according to the guidelines of the OECD guideline 221 [56]:

µ_i−j_ = [ln (N_j_) − ln (N_i_)]/7 (1)

In this equation, µ_ij_ accounts for the specific growth rate from i to j, N_i_ and N_j_ are the variables measured for each treatment (including the control) at time i (day 0) and j (day 7), and t is the time from i to j (7 days). The growth inhibition (Ir in%) was calculated using the Equation (2).

Ir = [(µ_C_ − µ_T_)/µ_C_] × 100 (2)

where µ_C_ and µ_T_ are, respectively, the specific growth rate for the control and treated sample.

### 3.7. Pigment Content and ROS Level

After 7 days of exposure, the remaining plants were grinded in a mortar containing 5 mL of 100% methanol at room temperature. The extract was then centrifuged at 1968× *g* for 5 min at 4 °C. Using a micro-plate reader (Infinite M200^®^, Tecan, Männedorf, Switzerland), the supernatant absorbance was measured at 665 nm (A_665_) for Chl *a*, and at 648 nm (A_648_) for Chl *b*. The pigment concentrations (µg mL^−1^) were estimated according to Equations (3) and (4) [57], and the ratio Chl *a*/*b* was determined based on fresh weight:

Chl *a* = 13.36 × (A_665_) − 5.19 × (A_648_) (3)

Chl *b* = 27.43 × (A_648_) − 8.12 × (A_665_) (4)

For the ROS level assessment in the cytosol of live cells, the plants were incubated for 30 min at 25 °C with the fluorogenic probe CellROX orange at 5 µM (absorption/emission maxima of 545/565; ThermoFisher Scientific, Waltham, MA, USA). After the incubation, the plants were washed with phosphate-buffered saline solution for 10 min at room temperature. The relative fluorescence emission was measured at 565 nm with a micro-plate reader (Infinite M200, Tecan) having a gain of 200 using a light excitation wavelength at 545 nm.

### 3.8. Statistical Analysis

For all toxicity tests, the means and corresponding standard deviations were determined for triplicate measurements (n = 3). The difference between treated and control (Blank) samples were tested using a one-way analysis of variance with Tukey’s post-hoc test for *p* < 0.05, using Graph Pad prism software version 8.0.2.

## 4. Conclusions

These results allow concluding that the pesticide toxicity towards *L. minor* evolves in time upon oxidation according to their molecular structure and the presence of clay minerals. *Lemna minor* turned out to provide accurate assessment of pesticide ecotoxicity. The latter affects the plant growth through excessive oxidative stress and changes in the distribution of the photosynthetic pigments. ROS level differs according to the pesticide concentration, molecular structure, degradation level, and action mechanisms. Massive adsorption of pesticides induced the appearance of diffusion hindrance but reduced the toxicity towards *L. minor*. High DAZ conversion by ozonation resulted in toxicity decay, more pronounced in the presence of Fe(II)-exchanged montmorillonite as compared to NaMt. The progressive appearance of more harmful ATR derivatives shades the simultaneous depletion of the parent molecule resulting in a constant toxicity in time. The toxicity towards *L. minor* and ROS level evolve simultaneously according to the catalytic activity of the clay minerals and during the progressive oxidative degradation of the pesticides and derivatives. A constant ROS level arises from enhanced pesticide conversion by ozonation and progressive rise of harmful derivatives. These results are of great importance because they provide valuable findings that allow predicting pesticide ecotoxicity according to its molecular structure, the type of clay-containing host-media and exposure time to ambient factors.

## Data Availability

The data generated during this study can be obtained upon request to the corresponding authors.
